# Shaolin Dan Tian Breathing Fosters Relaxed and Attentive Mind: A Randomized Controlled Neuro-Electrophysiological Study

**DOI:** 10.1155/2011/180704

**Published:** 2010-09-22

**Authors:** Agnes S. Chan, Mei-Chun Cheung, Sophia L. Sze, Winnie Wing-Man Leung, Dejian Shi

**Affiliations:** ^1^Neuropsychology Laboratory, Department of Psychology, The Chinese University of Hong Kong, New Territories, Hong Kong; ^2^Integrative Neuropsychological Rehabilitation Center, The Chinese University of Hong Kong, New Territories, Hong Kong; ^3^Henan Songshan Research Institute for Chanwuyi, Henan 452470, China; ^4^Institute of Textiles and Clothing, The Hong Kong Polytechnic University, Kowloon, Hong Kong

## Abstract

Neuro-electrophysiological studies on meditative breathing revealed its association with either a relaxed or an attentive state. The present study aimed to investigate whether the Shaolin Dan Tian Breathing (DTB) technique, which consists of the Passive and Active subtypes and can be considered as a relaxation exercise and Qigong, would induce both relaxed and attentive states. Twenty-two adults and 22 age-, gender- and education-matched controls received training on the Shaolin DTB (experimental group) and the progressive muscle relaxation respectively for one month. Eyes-closed resting EEG data before and immediately after each type of breathing were obtained individually at baseline and after one-month training. At baseline, the EEG changes after the Shaolin DTB between both groups were comparable. After one-month training, participants in the experimental, but not the control, group showed enhanced temporal alpha asymmetry (an index of relaxation and positive mood) after performing the Passive DTB for five minutes, and enhanced intra- and inter-hemispheric theta coherence (an index of attention and alertness) after performing the Active DTB. The present findings suggested a positive effect of the Shaolin DTB technique on enhancing human neural activity and connectivity, which may possibly enhance mood state and cognitive functions.

## 1. Introduction

Increasing empirical evidence has shown that mind-body training has positive effects on mental and physical problems such as depression [1–3], anxiety [4], obsessive-compulsive disorder [1, 5], insomnia [6], chronic pain [7], tension headaches [8], hypertension [9, 10], cardiac diseases [11], diabetes [12], irritable bowel syndrome [13], cancer [14], and viral infection [15]. Meditative breathing techniques, being a form of mind-body practice, have received increased interest for clinical use given that increasing empirical evidence suggests their positive effects on emotion [16–18], cognition [16, 19–22], and other physiological responses [23]. With the advanced development of tools to measure brain activation (i.e., EEG, fMRI), the effect of meditative breathing on brain activity has been recently revealed (see [24] for review), and increasing evidence is accumulating to suggest an association between the change of brain activities and meditative breathing practice. Specifically, Davidson and colleagues [25] demonstrated that there was an elevated hemispheric EEG asymmetry in the anterior brain region after an 8-week mindfulness-based stress reduction (MBSR) intervention of diaphragmatic breathing among meditators compared with controls. Other brain imaging studies have shown increased activation in various brain regions, including the prefrontal, temporal, parietal, and the anterior cingulate cortex while performing meditation [26–28].

Among the various types of breathing exercises, mindfulness meditative breathing is relatively common and has been more widely studied. Mindfulness meditative breathing involves a nonjudgmental observation of one's own breathing without reacting to or engaging in any thoughts. EEG studies reveal that this type of breathing generally induces a relaxation response, which can be indicated by an increase in alpha asymmetry in the anterior frontal and temporal regions between the two hemispheres of the brain [25, 29]. Greater left-sided activation, particularly in the anterior frontal and temporal regions, has been associated with reduced anxiety, increased positive mood, and the feeling of well-being [30–32].

While many neurophysiological studies on mindfulness-based breathing reported a change of brain activities that is associated with relaxation, some studies on concentrative meditation breathing (another form of meditation which requires a conscious focus of attention on the continuous rhythm of inhalation and exhalation) reported an enhancement of attention, as reflected by elevated EEG theta coherence [33, 34]. The theta coherence was found to be associated with sustaining and selective attention [35, 36] and working memory [37–41]. In sum, neuroelectrophysiological studies on various types of meditative breathing revealed that they are associated with either a relaxing state (i.e., enhanced alpha asymmetry in the anterior brain regions) or an attentive state (i.e., enhanced intra- and inter-hemispheric theta coherence).

On the other hand, it has been suggested that training that produces a relaxed and attentive brain state together will enhance the performance of individuals [42–45], for such a brain state enables individuals to get rid of distraction, remain focused on task, and cope with stressful work demand in a relaxed and flexible manner. The present study aimed to investigate whether the Shaolin Dan Tian Breathing can induce both the relaxed and attentive brain states. The Shaolin Dan Tian Breathing method used to be an in-house training in the Shaolin Temple and has been practised for over thousands of years in the Yonghuatang school of the Shaolin Temple. This type of breathing is different from commonly known breathing techniques in that it is also a form of Qigong. Qigong is a traditional Chinese mind-body training based on the fundamental concept of gathering and filling the Qi (bioenergy) inside the body, and the aim is to maintain mental and physical health. The Shaolin Dan Tian Breathing is a way to stimulate the Dan Tian region and to enhance the blood and Qi circulation. This technique consists of two components—Passive Dan Tian Breathing (Passive DTB) and Active Dan Tian Breathing (Active DTB). Initial fMRI data from four experienced practitioners showed that the two types of breathing activated overlapping and distinctive brain regions [46, 47]. While both activated the middle frontal and temporal gyri, the Passive DTB elicited unique activation in the cingulate gyrus and the Active DTB in the bilateral amygdala and parahippocampal gyri. Such findings suggested distinctive neural mechanisms underlying these two breathing techniques and possibly affecting different cognitive processes. The activated brain regions of these two DTB methods are crucial for mediating emotional, attentional, and memory processing.

Clinical observations [48] and empirical evidence [16, 49, 50] suggested that the Shaolin DTB has a beneficial effect on improving mood problems (e.g., depressive and anxiety mood) and cognitive problems related to brain disorders (e.g., epilepsy, autistic spectrum disorder). Specifically, a randomized controlled study has demonstrated the effect of one-month DTB training on reducing the depressive mood of community-dwelling adults [16]. An adolescent with Asperger's disorder demonstrated improved self-control of emotional outbursts, repetitive behaviors, and problem-solving abilities after three months of DTB training [49]. A patient suffering from chronic epilepsy and stroke showed improved short-term memory, expressive and comprehensive abilities after a four-month intervention with the Shaolin DTB as one of the treatment components [50].

**Figure 1:**
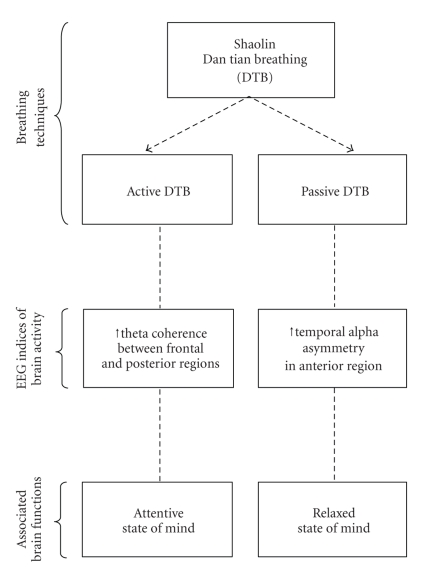
Schematic representation of the effect of Shaolin Dan Tian Breathing (DTB) on the states of the mind.

Our clinical observation and fMRI results suggested that the Shaolin DTB technique has positive effects on mood as well as cognitive functions. The present study utilizes two quantitative EEG measurements, namely, alpha asymmetry and theta coherence, to examine the electrophysiological state of the brain after an individual practised this unique breathing method. While alpha asymmetry in the anterior brain regions was found to be associated with a relaxed mind, theta coherence was found to be associated with attentiveness. It was hypothesized that the Passive DTB would induce the relaxation response as reflected by an increase in temporal alpha asymmetry in the anterior brain region while the Active DTB would increase attention as indicated by elevated theta coherence, particularly in the connections between frontal and posterior brain regions. A schematic representation of the effect of Shaolin DTB is shown in Figure 1.

## 2. Subjects and Methods

### 2.1. Participants

Fifty community-dwelling adults who were recruited from a public lecture on mental health awareness participated voluntarily in the study. All participants reported no history of head injury, seizure, stroke, other CNS diseases, or psychiatric illnesses of psychosis or mania. The participants were informed that they would be randomly assigned to one of two groups, where different training techniques would be taught with the aim to study the effects of the two relaxation methods. They were blind to the potential benefits of the two techniques and had no information on the technique practised by the other group. Equal numbers of participants were randomly assigned using the block randomization method into the experimental group which received training on the Shaolin DTB techniques and the control group which received training on progressive muscle relaxation. Throughout the four weekly 90-minute training sessions (6 hours of training within one month), both groups were taught to master the respective techniques and were monitored on their practice by experienced clinical practitioners, who were not involved in EEG data collection. Assessment of the participant's EEG and bio-physiological indices was administered at baseline and after training. Eight participants (3 in the experimental group, 5 in the control group) were excluded from the study for low attendance in the sessions (under 75% of the training sessions) or absence from the postassessment, resulting in a total of 22 participants in the experimental group and 20 participants in the control group. The participants were aged 25 to 64 years and had attained at least 9 years of education. The two groups were matched on age (*t*_(40)_ = 0.32, *P* = .75), education level (*t*_(40)_ = 0.15, *P* = .88), and gender (*χ*_(1)_^2^ = 0.03, *P* = .87). Demographic characteristics of the participants are shown in Table 1.

### 2.2. Procedure

The study was conducted in accordance with the Helsinki Declaration of the World Medical Association Assembly. The experimental procedure was approved by the Joint CUHK-NTEC Clinical Research Ethics Review Committee. Informed consent was obtained from all participants. EEG and bio-physiological data were recorded simultaneously from each participant in a sound- and light-attenuated room at baseline and after the one-month training. At baseline, both groups of participants had no experience of DTB. During the one-month training, while the experimental group received training on the DTB techniques, the control group received training on progressive muscle relaxation. At each measurement time point, participants began with a 5-minute eyes-closed resting condition. Then, they were required to perform 5-minute Passive DTB, followed by a 5-minute rest. After that, participants were asked to perform a 5-minute Active DTB, and again followed by a 5-minute rest. EEG and bio-physiological data were obtained during the three resting conditions: (1) prebreathing rest; (2) post-Passive DTB rest, and (3) post-Active DTB rest, which were then used for subsequent analyses. 

For the EEG recording, each participant was hooked up using the TruScan measuring set with 19 electrodes positioned across the scalp according to the International 10–20 System [51]. All electrode impedances were kept at 10 k*Ω* or below. The EEG signals were referenced to linked ears and digitally filtered at 0.5 and 100 Hz and sampled at 256 samples per second, with a high-frequency limit band pass of 30 Hz. Artifacts were removed offline based on records of bodily movements of the participant by the experimenter throughout the EEG assessment. 

Simultaneous measurements of bio-physiological data including heart rate (HR), respiratory rate (RR), and skin conductance (SC) were obtained during EEG recording, using the ProComp Infiniti system, and analyzed with the BioGraph Infiniti software manufactured by Thought Technology Ltd (2005) [52]. HR, RR, and SC have been reported to be sensitive measures of the meditation-induced relaxation state, and were thus used to reflect sympathetic arousal levels (see [53] for a review). HR was calculated from the blood volume pulse (BVP) signal, which is an indicator of blood flow resulting from each heart beat. The BVP senor was attached to the palmar side of the left thumb, which took measurement at 256 samples/second. The RR and SC levels were measured at 32 samples/second. The RR was measured by placing the sensor over the diaphragm with a large Velcro belt extending around the chest cavity. While the participant's chest cavity expanded during inhalation, a small elastic band attached to the sensor stretched, from which the rate of respiration was measured. In measuring the SC, the sensor was attached to the left index and ring fingers with Velcro. 

### 2.3. Dan Tian Breathing and Progressive Muscle Relaxation

Throughout the one-month training period, the experimental group received training on the Shaolin DTB delivered by the first author whereas the control group was trained on progressive muscle relaxation by a clinical psychologist who has over 10 years of clinical experience and is working in a public hospital. He frequently applied this technique in his therapy sessions for the hospital patients. This trainer is affiliated neither with the Chanwuyi Foundation nor the Henan Songshan Research Institute for Chanwuyi. Furthermore, in order to reduce bias, both training groups were conducted in a rented room of the Young Men's Christian Association (YMCA) instead of either The Chinese University of Hong Kong or the public hospital, in order to minimize any association with the training techniques the participants might have. Participants learnt the techniques as well as their fundamental principles during the first training session. They were requested to practise the techniques at home regularly and were monitored in the subsequent three weekly training sessions. In order to ensure that the participants practised regularly at home, a log record of daily practice frequency and duration was required. In order to ensure the quality of practice of techniques for both groups, the respective trainers observed each participant during each training session to make sure the techniques that the participant practised were correct.

The Shaolin DTB consists of two forms: passive and active. Both forms begin with placing the hand(s) gently onto the Dan Tian region (i.e., half an inch below navel). While the Passive DTB was performed by passively observing the Dan Tian region when inhaling and the nose when exhaling with eyes closed, the Active DTB required the tightening of the anal and abdominal muscles with closed lips and naturally opened eyes during exhalation and total relaxation with closed eyes when inhaling. Participants were instructed to practise both forms alternately within one session. They were required to stop when he/she felt relaxed and/or felt a bit warm. Thus, the duration of practice varied from person to person.

Progressive muscle relaxation is a stress management technique developed by Jacobson [54], which is a widely practised relaxation method and has been reported to be effective in alleviating anxiety and mood problems [55]. Participants were taught according to the standard procedure of the technique: beginning with some deep breaths with closed eyes, followed by alternately tensing and relaxing groups of muscles in a prescribed sequence from the head down along the chest, the hands, and eventually the feet. Participants were to inhale when tensing the muscles, exhale when relaxing, and feel the bodily changes throughout the tensing/relaxing cycle.

### 2.4. EEG Data Analyses

#### 2.4.1. Temporal Alpha Asymmetry

Data of absolute power at the alpha frequency band (8–13 Hz) were first selected and then normalized with natural logarithm (ln) transformation. The alpha asymmetry in the anterior temporal (T3 and T4) region was used given its reported positive association with positive mood and relaxed mind [25, 56]. It is calculated as [ln(T4)−ln(T3)], where T4 is the alpha power on the right hemisphere and T3 on the left. Since alpha power was an inverse measure of brain activity, positive temporal alpha asymmetry values resulting from higher right-alpha power indicated greater left relative to right brain activity (i.e., left-sided activation) whereas negative asymmetry values indicated right-sided activation.

#### 2.4.2. Theta Coherence

EEG coherence, with values ranging from 0 to 1, is a functional cooperation index measuring the temporal synchronization between signals at two electrode sites as a function of frequency [57, 58]. It provides useful information about underlying cortical coupling and connectivity between distinct brain regions [59, 60]. High EEG coherence indicates strong structural or functional connection between cortical regions [61, 62] and is associated with various cognitive tasks such as attention [35, 36], memory [40, 59, 63], language [60, 64], and intelligence [65]. Coherence can be classified as short range and long range. While short-range intrahemispheric coherence refers to the synchronization between adjacent electrodes to tap the connectivity within the same hemisphere, long-range coherence refers to those obtained from any electrode pairs that are separated by at least one electrode in between and reflects the connectivity between two distal regions within or between hemispheres. The coherence values at theta band (3–7 Hz) measured at the intra- and inter-hemispheric connections within and between the frontal and posterior scalp regions were obtained, and its square rooted values were then normalized using Fisher's *Z* transformation. The transformed coherence values were grouped into six connectional pairs: short-range intra-hemispheric coherence in the (i) frontal (F3–F7 and F4–F8), (ii) posterior (P3-O1 and P4-O2) cortical regions, (iii) long-range intrahemispheric coherence in the frontoposterior regions (F3-P3, F4-P4, F7-P3, F8-P4, F3-O1, F4-O2, F7-O1, and F8-O2), and long-range inter-hemispheric coherence in the (iv) frontal (F3-F4, F7-F8, F3-F8, and F4-F7), (v) posterior (P3-O2, P4-O1, P3-P4 and O1-O2), and (vi) fronto-posterior regions (F3-P4, F4-P3, F7-P4, F8-P3, F3-O2, F4-O1, F7-O2, and F8-O1). The specific electrode sites were chosen and grouped based upon previous studies reporting that short-range and long-range frontal and posterior theta coherence indices are sensitive to attentional processes [35, 36]. 

### 2.5. Bio-Physiological Measures

The HR, RR, and SC data collected were averaged using the BioGraph Infiniti software every minute. The mean values were computed for each 5-minute session before, during, and after the Shaolin DTB at baseline and after training.

### 2.6. Change Indices

The change indices represent the change in EEG and bio-physiological states before and after performing the Shaolin DTB at each evaluation (i.e., at baseline and after training). For instance, the Passive DTB-induced asymmetry change at baseline is calculated as (post- minus pre-Passive DTB at baseline). The change indices were used for between-group comparisons before and after the one-month training to evaluate training effect of both the Passive and Active DTB on brain activity and bio-physiological level.

## 3. Results

### 3.1. Passive Dan Tian Breathing Enhanced Temporal Alpha Asymmetry

**Figure 2:**
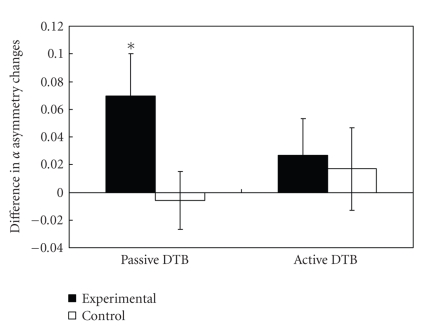
Comparison of the temporal alpha asymmetry between the experimental and control groups. Each bar represents the difference between baseline and posttraining values, where positive values indicate increased breathing-induced temporal alpha asymmetry and negative values indicate a reduction in asymmetry. *Paired sample *t* statistics with *P* < .05.

Separate repeated-measures ANOVA on temporal alpha asymmetry change was performed for each type of Dan Tian Breathing (Passive versus Active), with time (baseline versus after one-month training) as within-subject factor and group (experimental versus control) as between-subject factor. The multivariate results showed a marginally significant interaction effect of time and group (*F*_(1,40)_ = 4.05, *P* = .05, partial *η* ^2^ = 0.09, large effect size). Post hoc paired *t*-tests showed significant Passive DTB-induced elevation in temporal alpha asymmetry after one month of training in the experimental (baseline: mean ± SD = −0.018 ± 0.065; after training: mean ± SD = 0.052 ± 0.101; *t*_(21)_ =2.31, *P* = .03) but not the control group (baseline: mean ± SD = 0.020 ± 0.055; after training: mean ± SD = 0.014 ± 0.077; *t*_(19)_ = −0.27, *P* = .79). In contrast to the Passive DTB, the multivariate results showed that both groups did not show significant changes in asymmetry index after performing the Active DTB (*F*_(1,40)_ = 1.20, *P* = 0.28).  Figure 2 shows the difference in the DTB-induced temporal alpha asymmetry change at baseline and after training. 

It should be noted that at baseline, the Passive DTB-induced temporal alpha asymmetry change was comparable between the two groups (*t*_(40)_ = 2.01, *P* = .051). Both experimental (pre-breathing: mean ± SD = 0.186 ± 0.195; post-Passive DTB: mean ± SD = 0.168 ± 0.204; *t* = −1.59, *P* = .13) and control (pre-breathing: mean ± SD = 0.206 ± 0.206; post-Passive DTB: mean ± SD = 0.226 ± 0.232; *t* = 1.30, *P* = .21) groups did not show significant enhancement in temporal alpha asymmetry after the 5-minute practice of the Passive DTB. Therefore, the temporal alpha asymmetry increment observed in the experimental group after the one-month training suggested that the Passive DTB had a significant effect on brain activity, possibly associated with a more relaxed state of mind. 

### 3.2. Active Dan Tian Breathing Enhanced Theta Coherence

**Figure 3:**
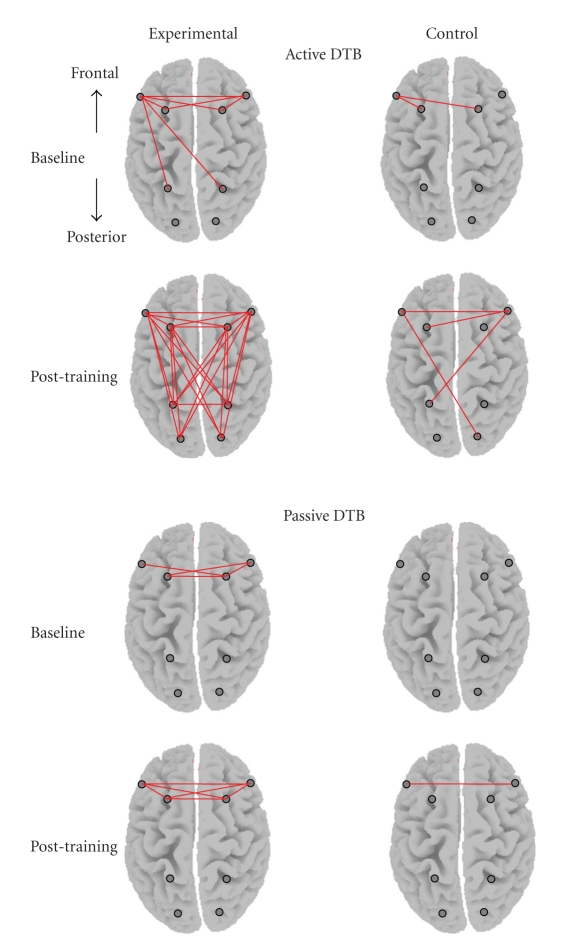
Topographic maps demonstrating the change indices of theta coherence at baseline and after one-month training for the experimental and control groups. Straight lines linking the electrode pairs signify significant breathing-induced coherence increments analyzed with paired *t* tests, *P* < .05.

The topographic presentation in Figure 3 shows the theta coherence change indices of the 28 individual coherence pairs composing the six groups of regional connections within and between the frontal and posterior areas. Straight line linking between electrodes (indicated as dots on the scalp) indicates significant increase in coherence analyzed by paired *t*-tests, *P* < .05. The results showed that after the one-month training, the experimental group demonstrated significantly enhanced theta coherence across the majority (82%) of the electrode pairs connecting cortical regions within and across hemispheres after the Active DTB practice whereas the control group showed substantially fewer connections (at most 25%). Further analyses were performed on the mean coherence values at the three regional connections across conditions between groups. 

**Figure 4:**
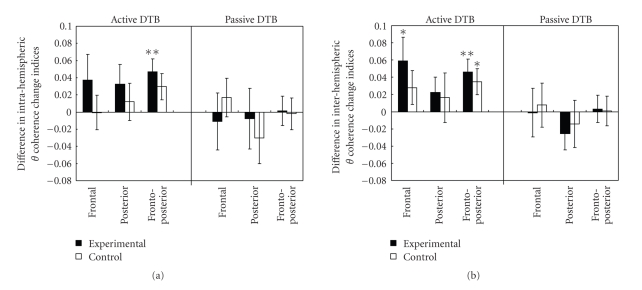
Difference in (a) intra-hemispheric and (b) inter-hemispheric theta coherence change indices at baseline and after one-month training for the experimental and control groups. Positive values indicate increased breathing-induced theta coherence changes after one-month training; negative values indicate a reduction in theta coherence. Paired sample *t*-tests with *P ≤ .05; **P < .01.

Separate 2 × 3 × 2 repeated-measures ANOVA was performed on intra- and inter-hemispheric theta coherence to compare pre- and posttraining (time: baseline versus after one-month training) changes on three regional connections (region: frontal versus posterior versus fronto-posterior) between the two groups (group: experimental versus control). For intra- and inter-hemispheric theta coherence changes associated with the Active DTB, the multivariate test results showed a significant main effect of time (intra: *F*_(1,40)_ = 6.59, *P* = .01; inter: *F*_(1,40)_ = 9.66, *P* = .003), and between-subject difference (intra: *F*_(1,40)_ = 5.24, *P* = .027; inter: *F*_(1,40)_ = 5.55, *P* = .023), respectively. The main effect of time was further tested within each group with repeated measures ANOVAs (time by region) and post hoc paired *t*-tests. The multivariate test results indicated a significant main effect of time, where the experimental group showed greater increase in the intra-hemispheric theta coherence (*F*_(1,21)_ = 7.32, *P* = .01) and inter-hemispheric theta coherence (*F*_(1,21)_ = 7.55, *P* = .01) after the one-month training, but the control group did not show such significant increment (intra: *F*_(1,19)_ = 0.89, *P* = .36; inter: *F*_(1,19)_ = 2.78, *P* = .11). As indicated by the post hoc *t*-tests, the significant increment demonstrated in the experimental group was primarily in the intra- and inter-hemispheric frontal-posterior connections (intra: *t*_(21)_ = 3.12, *P* = .005, medium effect size of 0.67; inter: *t*_(21)_ = 3.05, *P* = .006, medium effect size of 0.65) and inter-hemispheric frontal connections (*t*_(21)_ = 2.16, *P* = .04, small effect size of 0.46) (Figure 4) while the control group demonstrated increment only in the inter-hemispheric frontal-posterior connections (*t*_(19)_ = 2.30, *P* = .03, small effect size of 0.52). It should be noted that all theta coherence change indices at baseline in the experimental group were comparable to those of the control group, with t-values ranging from 0.37 to 2.0, *P* > .05. For the Passive DTB, as anticipated, results of repeated-measures ANOVA did not show any significant alteration in intra- or inter-hemispheric theta coherence in either group (intra: *F*_(1,40)_ = 0.22, *P* = .64; inter: *F*_(1,40)_ = 0.21, *P* = .65). 

Overall, the findings were consistent with our hypothesis that the two types of the DTB have dissociable effects on different mental states. While the Passive DTB was found to be associated with the relaxed state of mind as reflected by the enhanced temporal alpha asymmetry, the Active DTB was found to be associated with the attentive state of mind as reflected by the enhanced theta coherence, particularly in the intra- and inter-hemispheric frontal-posterior connections. Furthermore, the changes in brain activities were more pronounced in the experimental group than the control group.

### 3.3. Association of Neurophysiological Changes with Practicing Habits

Given that participants were instructed to stop practising the two DTB techniques when they felt relaxed and/or warm in the body, the duration of practice varied among participants as some reached the state after a few minutes while others needed more time. It was thus hypothesized that the changes in EEG activities would not be strongly associated with the duration of practice. Correlation analyses within the experimental group were performed to explore this issue. Experimental group participants kept a log record of their daily practice frequency and duration throughout the one-month training period. For the Passive DTB, the mean duration and frequency of practice was about 13 minutes twice per day (minimum: 3 minutes once per day; maximum: 30 minutes 5 times per day). For the Active DTB, the mean duration and frequency of practice was about 6 minutes once per day (minimum: 3 minutes once per week; maximum: 15 minutes thrice per day). The intensity of practice, in terms of total frequency and duration in a month, was computed separately for the two forms of the DTB. Then, the association between the intensity of practice and the EEG change indices before and after the training was analyzed using Pearson's correlation. There was no significant correlation (*P* > .05) between the intensity of practice of the Passive DTB and changes in temporal alpha asymmetry (total frequency: *r* = 0.10; total duration: *r* = 0.17) and between the intensity of practice of the Active DTB and changes in theta coherence (total frequency: *r* ranges from −0.17 to 0.22; total duration: *r* ranges from −0.26 to 0.09) meaning that increment in brain activity was not linearly associated with practice intensity of DTB.

### 3.4. Calming Effect of Dan Tian Breathing on Autonomous Nervous System (ANS)

Many past studies measured the calming effect of meditative breathing on ANS by means of bio-physiological indices, with the majority of these studies showing either biophysiological changes during breathing processes, or performance at base rate of experienced practitioners. The pattern of the autonomic effect of DTB found in the present study was generally consistent with past findings [53, 66–68]. Significant reduction of respiration rate was observed during Passive DTB (*t*-values ranging from 2.95 to 8.80, *P* < .05) and Active DTB (*t*-values ranging from 2.34 to 6.77, *P* < .05), compared with the prebreathing resting states in both groups at baseline and after training (reduced by 3 to 5 breaths/min). On average, the individuals took 8 to 10 breaths per minute while performing the DTB, with each cycle of inhalation and exhalation lasting for about 6 to 8 seconds. This means that both forms of the DTB were able to slow down the respiration rate during the breathing process. When comparing the respiration rate at pre-breathing resting state at baseline and that after one-month training (i.e., the base rate changes before and after one-month training), the experimental group did not show significant changes (*t*_(21)_ = 1.78, *P* = .09) but the control group showed a significant increase in respiration rate (*t*_(19)_ = 2.33, *P* = .03). For the comparison on the base rate of heart rate at pre- and post-one-month training, the experimental group showed a trend of reduction in heart rate (by 3.63 beats/min, *t*_(21)_ = 1.96, *P* = .06) whereas the control group did not (by 2.47 beats/min, *t*_(19)_ = 1.22, *P* = .24). However, there was no significant change in the SC index before and after training (*t* ranges from 0.66 to 1.60, *P* > .05) in both experimental and control groups. 

## 4. Discussion

The present study has demonstrated the neuro-electrophysiological effects of the Shaolin Dan Tian Breathing (DTB) after one month of training. The results showed that participants in the experimental but not the control group showed enhanced temporal alpha asymmetry and theta coherence levels after the one-month practice, with a mean of 15 minutes a day. These results suggested that practicing the Shaolin DTB facilitated the attainment of the coexisting states of relaxed and attentive mind, which made this breathing technique uniquely different from other more well-known breathing techniques. Specifically, the majority of electrophysiological studies on meditative breathing reported either elevated anterior alpha asymmetry [16, 25, 29] or theta coherence [33, 34] but rarely both [22]. The Shaolin DTB technique, on the other hand, seemed to be able to enhance both temporal alpha asymmetry and theta coherence, suggesting that this technique may be useful to induce a relaxed and attentive mind state. 

These results are consistent with the clinical observation of individuals who have practised this method as complementary intervention for their emotional and cognitive deficits. For instance, a randomized controlled study has demonstrated the effect of one-month DTB training on reducing the depressive mood of community-dwelling adults [16]. An adult suffering from anxious mood and overwhelming stress reported to experience emotional calmness and reduced anxiety after practicing the Shaolin DTB for two months [48]. An adolescent with Asperger's disorder practicing the Shaolin DTB for three months showed improved inhibition of inappropriate behaviors and emotions and cognitive flexibility [49]. Another study also provided evidence to suggest the positive effects of the Shaolin DTB on a patient with epilepsy and stroke (e.g., improved short-term memory, expressive and comprehensive abilities) [50]. Thus, while some behavioral studies suggested that the Shaolin DTB may have a therapeutic effect on emotional and cognitive dysfunctions, the present study has provided some empirical data to explain the possible neural mechanisms underlying the therapeutic effects. 

The principle for the development of the Shaolin DTB was based upon Chinese martial arts and Qigong that cannot be fully explicable using western concepts. Specifically, the Shaolin DTB consists of two forms, Passive and Active, that need to be practised together alternately, for instance, practicing five times Passive DTB and one time Active DTB, or ten times Passive DTB and three times Active DTB (the frequency depends on the individual's feeling). The rationale for practicing the DTB this way was to enhance the blood and Qi circulation more effectively. Thus, the Shaolin DTB is not simply a relaxation exercise but also a form of Qigong. Results from the present study showed that practicing the DTB induced not only a relaxed (i.e., temporal alpha asymmetry) but also an attentive (i.e., theta coherence) state of mind, providing some pilot data to support the rationale that the Shaolin DTB is more than a relaxation exercise.

Some empirical studies have suggested that enhancing both a relaxed and an attentive mind states is crucial for achieving higher cognitive performance, such as enhanced attention and concentration, faster reaction time, better decision making ability, and increased creativity, and thereby improving work efficiency and productivity [42, 45]. One exploratory method of performance enhancement training is through neurofeedback training. Its assumption is based on the association between particular EEG patterns of cortical activity and specific optimal states of cognitive or psychological function. Through neurofeedback, the individual learns to modify his/her cortical activity and activates the EEG patterns seen during optimal states, and a typical training usually takes 10 to 20 sessions, or even more [42, 44]. Some empirical studies on performance enhancement training through neurofeedback showed that increase in EEG alpha amplitude in the anterior brain regions and synchronization can improve sustained attention, creativity, problem-solving ability, task accuracy, and work productivity [42, 44, 69, 70]. While the present study demonstrated that one-month DTB training had similar effects as performance enhancement training through neurofeedback (i.e., increasing temporal alpha asymmetry and theta coherence), the Shaolin DTB has advantages over training through neurofeedback in that it is less costly and time consuming. Therefore, there may be potential for the Shaolin DTB to be developed as a way to enhance human cognitive functions and performance.

The majority of the studies on meditative breathing reporting positive changes in brain activity usually recruited practitioners with at least one year or even up to more than 20 years [71–73] or provided intensive/long-term intervention programs for novice practitioners to elicit the positive effects. For example, the extensively researched Transcendental Meditation Program requires 10 hours of lecture, and the Mindfulness-Based Stress Reduction program requires about 30 hours. Some of them failed to find positive effect among novice practitioners who have no experience or have practised for less than a year [33, 34]. It should be noted that the participants in the present study were all beginners with no previous experience in the DTB, and the training period was as brief as four weekly sessions over a month. Moreover, the participants have practised on average for only about 15 minutes per day. Thus, it may be a relatively easy-to-learn and easy-to-practise method. Nevertheless, the present study has only provided evidence for the immediate effect of one-month DTB training. It is worth exploring in future studies the long-term effect of the breathing techniques.

Furthermore, the treatment effect of the Shaolin DTB may be clinically significant. Since the Shaolin DTB is easy to learn and practise, does not need any equipment to facilitate the training, and is cost-effective, it is worth examining its efficacy in treating clinical populations with neurological and psychiatric disorders, especially those with attention or memory deficits, anxiety or mood problems, or patients who are wheelchair or bed bound. In addition, in view of the findings of suppressed anterior alpha asymmetry in patients with depression [74–76] and reduced fronto-posterior theta coherence in patients with Alzheimer's disease [77, 78] and schizophrenia [79], the Shaolin DTB, with its induced temporal alpha asymmetry and theta coherence elevation, may be applicable as a complementary intervention for normalizing brain electrophysiological abnormalities of these patients to improve their cognitive and mental abilities. 

The present study has provided encouraging evidence on the positive neuro-electrophysiological effects of the Shaolin DTB technique after one month of training, by inducing a coexisting brain state of relaxation and attentiveness that is crucial for achieving peak performance. It should be noted, however, that the present study assessed only the physiological indices of the attentive and relaxed state and did not include psychological measurements or other measures of the subjective emotions and cognitions of the participants to supplement the objective physiological findings. Future studies should include this information to supplement the objective physiological measures. In spite of this limitation, given the easy-to-practise and cost-effective characteristics of the Shaolin DTB and its potential effect on enhancing brain activity, the clinical applicability of this technique as a complementary intervention for patients with brain disorders has been implicated.

## Figures and Tables

**Table 1:** Demographic characteristics of the participants.

	Experimental group (*n* = 22)		Control group (*n* = 20)	
	Mean	SD	Mean	SD
*Age *(years)	49.66	6.92	48.92	8.11
*Education *(years)	12.73	2.66	12.85	2.72
*Gender—Female *(%)	75		73	
